# Subgingival Microbiome in Rheumatoid Arthritis Patients with Periodontitis

**DOI:** 10.3390/ijms23179883

**Published:** 2022-08-31

**Authors:** Yi-Jing Chen, Wei-Chun Hung, Yu-Hsiang Chou, Chern-Hsiung Lai, Po Peng, Pei-Syuan Jhou, Min-Ru Tsai, Jim Jinn-Chyuan Sheu, Jeng-Hsien Yen

**Affiliations:** 1 Department of Microbiology and Immunology, School of Medicine, College of Medicine, Kaohsiung Medical University, Kaohsiung 807378, Taiwan; 2 Division of Periodontics, Department of Dentistry, Kaohsiung Medical University Hospital, Kaohsiung 807377, Taiwan; 3 School of Dentistry, College of Dental Medicine, Kaohsiung Medical University, Kaohsiung 807378, Taiwan; 4 Anaerobic and Oral Microbiology Testing Center, Kaohsiung Medical University, Kaohsiung 807378, Taiwan; 5 Department of Biomedical Science and Environmental Biology, Kaohsiung Medical University, Kaohsiung 807378, Taiwan; 6 Institute of Biomedical Sciences, National Sun Yat-sen University, Kaohsiung 804201, Taiwan; 7 Department of Biotechnology, Kaohsiung Medical University, Kaohsiung 807378, Taiwan; 8 Division of Rheumatology, Department of Internal Medicine, Kaohsiung Medical University Hospital, Kaohsiung 807377, Taiwan; 9 Graduate Institute of Clinical Medicine, College of Medicine, Kaohsiung Medical University, Kaohsiung 807378, Taiwan

**Keywords:** rheumatoid arthritis, subgingival microbiome, periodontitis, microbial dysbiosis, anti-citrullinated protein antibody (ACPA)

## Abstract

Rheumatoid arthritis (RA) and periodontitis are suggested to be closely linked based on microbial dysbiosis, but limited subgingival bacteria have been proven in the pathogenesis of RA. We enrolled 30 RA patients and 25 controls and divided them into three groups with matched age, gender, and diabetes statuses: group AM (all of the matched participants), group PD (periodontally diseased), and group PH (periodontally healthy). Their subgingival microbial composition was determined by V3–V4 16S rRNA gene sequencing. Significant differences in subgingival microbial clustering between the RA patients and controls were observed in groups AM and PD. Among the taxa enriched in RA, *Aminipila butyrica* and *Peptococcus simiae* were the only two species displaying positive correlation to the level of anti-citrullinated protein antibodies (ACPAs) in both of the groups. Surprisingly, the median of relative abundances of *A. butyrica* and *P. simiae* were 0% in the controls of group PD. Furthermore, a gene encoding arginine deiminase with the capability to produce citrulline was addressed in the complete genome sequence of *A. butyrica*. This is the first study to elucidate the important roles of *A. butyrica* and *P. simiae* as periodontal bacteria leading to RA possibly through the induction of ACPA production.

## 1. Introduction

The factors related to the pathogenesis of rheumatoid arthritis (RA), such as the genetic backgrounds, hormones, smoking, environmental factors, and subgingival microbes, have been widely studied, but their definite mechanisms remain to be explored. Epidemiological data have suggested a connection between RA and periodontitis, which is linked by periodontal pathogens. Recent studies have shown that *Porphyromonas gingivalis* (*P. gingivalis*), one of the red bacterial complex resulting in severe periodontitis, is closely related to RA. It expresses peptidyl arginine deiminase (PAD), which can catalyze the citrullination of proteins in both a host and bacteria without calcium [1,2]. The citrullinated proteins will be involved in the breakdown of immune tolerance to host molecules and finally cause an autoimmune response in RA patients. Leukotoxin A (LtxA) of *Aggregatibacter actinomycetemcomitans* (*A. actinomycetemcomitans*) is another etiological factor to produce citrullinated proteins. Lysis of the neutrophils by LtxA induces the expression of human PAD2/4 to catalyze hypercitrullination of proteins [2,3].

It has been found that people who are anti-citrullinated protein antibody (ACPA)-positive appear to be at an increased risk of developing RA [4]. The citrullinated proteins produced by periodontal pathogens were thought to be capable of inducing the production of ACPAs [2]. *P. gingivalis* has been found to have significant higher relative abundance in healthy periodontal sites in ACPA-positive individuals [5]. Another meta-analysis showed a significantly increased level of anti-*P. gingivalis* antibodies in RA patients who were ACPA-positive [6]. However, neither *P. gingivalis* nor *A.* *actinomycetemcomitans* was related to the alveolar bone loss or severity of arthritis in a mouse model [7]. It is suggested that persistent bacterial translocations from the oral cavity to the gut would increase intestinal permeability, resulting in penetration of oral bacteria into blood, triggering synovial autoimmunity in RA [8]. However, *P. gingivalis* might hardly survive the trip to the gut in humans [9] although it could translocate from a gingival site to stool in a mouse model of arthritis [10]. Therefore, it was proposed that other oral microbes might be associated with the pathogenesis and progression of RA [8].

Introduction of 16S rDNA sequencing on subgingival microbiota in periodontitis and RA revealed that the composition of subgingival microbiota could vary with the periodontal status or sampling on diseased/healthy sites from the same person [11,12,13]. Therefore, we designed a cross-sectional study to compare the subgingival microbial composition between the RA patients and controls with or without periodontitis.

## 2. Results

### 2.1. Grouping of the Participants and Comparisons of Clinical Characteristics

**Figure 1:**
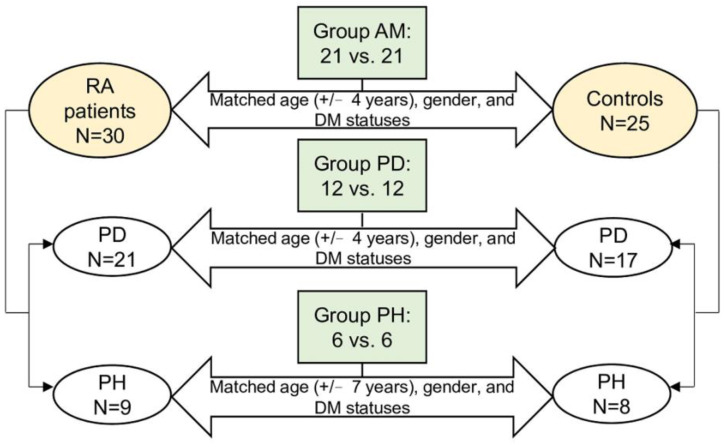
Study design and the flowchart. A total of 42, 24, and 12 participants were eligible for the final analysis in groups AM, PD, and PH, respectively. The RA and control participants were matched on age, gender, and DM statuses.

A total of 55 individuals, including 30 RA patients and 25 controls, were enrolled in this study. In the all matched (AM) group, the RA patients and controls were matched with age (+/−4 years), gender, and DM statuses regardless of their periodontal conditions. Finally, there were 42 individuals in group AM, of which 21 have RA, and 21 are controls (Figure 1). In the 30 RA patients, 21 individuals had periodontitis (PD), and nine individuals were periodontally healthy (PH). As for the 25 controls, 17 individuals had PD, while 8 individuals were PH. Thus, groups PD and PH included participants with and without periodontitis, respectively, and the RA patients and controls within each group were matched with the criteria as described in Figure 1. Finally, a total of 24 and 12 individuals were enrolled in groups PD and PH, respectively, and the RA patients and controls were half and half in each group. The presence of rheumatoid factor (RF) and ACPAs and the levels of ACPAs showed significant differences between the RA patients and controls in groups AM and PD (Table 1). Detailed information of each participant is shown in Supplementary Table S1.

### 2.2. The Alpha Diversity of Subgingival Microbiota between the RA Patients and Controls in the Three Groups

**Figure 2:**
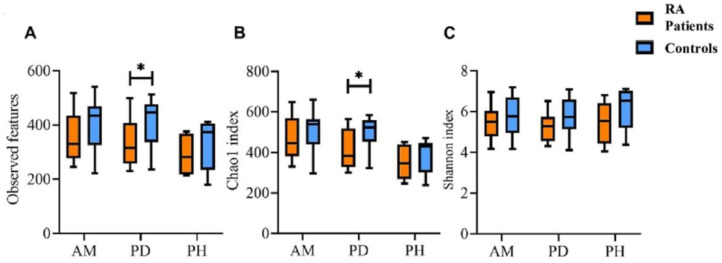
The intra-group diversity of subgingival microbiota in groups AM, PD, and PH. (**A**) The number of the observed bacterial features, (**B**) Chao1 index, and (**C**) Shannon index. The RA patients are indicated in orange, and the controls are indicated in blue. The data are expressed as median (25th, 75th percentile). *, *p* < 0.05.

The alpha diversity of microbial communities in groups AM, PD, and PH was evaluated by the number of the observed bacterial features, Chao1 index, and Shannon index. For the number of the observed bacterial features between the RA patients and controls, a significant difference was found in group PD only (334.7 ± 89.2 vs. 414.6 ± 87.0, *p* = 0.0375, Figure 2A). Similar results were observed in the Chao1 index, with significant decrease in the RA patients compared with that in the controls (416.2 ± 95.7 vs. 502.1 ± 74.8, *p* = 0.0496, Figure 2B). In contrast to the number of the observed bacterial features and Chao1 index (the community richness), no significance was observed in the Shannon’s index (the community evenness) between the RA patients and controls in the three groups (Figure 2C).

### 2.3. The Beta Diversity of Subgingival Microbiota between the RA Patients and Controls in the Three Groups

**Figure 3:**
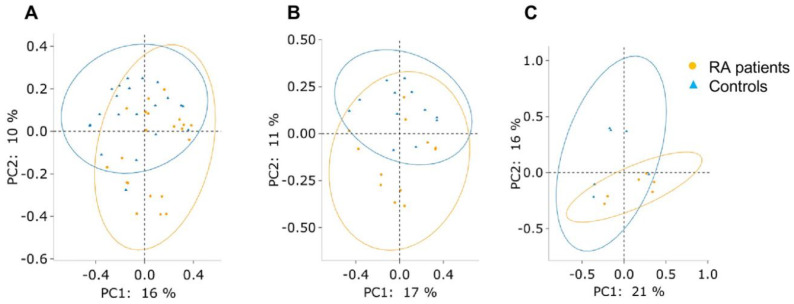
The subgingival microbiota clustering by PCoA based on Bray–Curtis dissimilarity between the RA patients (orange dots) and controls (blue triangles). (**A**) Group AM (ANOSIM: R = 0.177, *p* = 0.001 and PERMANOVA: pseudo-F = 2.561, *p* = 0.001), (**B**) group PD (ANOSIM: R = 0.15, *p* = 0.013 and PERMANOVA: pseudo-F = 1.722, *p* = 0.018), and (**C**) group PH (ANOSIM: R = 0.167, *p* = 0.116 and PERMANOVA: pseudo-F = 1.449, *p* = 0.076).

The PCoA based on Bray–Curtis dissimilarity displayed a significant difference in subgingival microbial clustering between the RA patients and controls in groups AM (ANOSIM: R = 0.177, *p* = 0.001 and PERMANOVA: pseudo-F = 2.561, *p* = 0.001) and PD (ANOSIM: R = 0.15, *p* = 0.013 and PERMANOVA: pseudo-F = 1.722, *p* = 0.018) but not in group PH (Figure 3). Therefore, group PH was excluded for further analyses because of the lack of a distinct pattern of subgingival microbiota between the RA patients and controls.

### 2.4. The Discriminative Taxa of the Subgingival Microbiota between the RA Patients and Controls in Groups AM and PD

The differences regarding the composition of subgingival microbiota between the RA patients and controls in groups AM and PD were estimated by LEfSe with the parameter of the logarithmic LDA score > 2. In group AM, 176 discriminative taxa in total were found (Supplementary Figure S1A). Of the species level with the logarithmic LDA score > 4, *Streptococcus anginosus* (*S. anginosus*), *Treponema denticola* (*T. denticola*), and an unidentified *Fusobacterium* species were enriched in the RA patients, while *Haemophilus parainfluenzae* *(H. parainfluenzae*) and *Streptococcus sanguinis* (*S. sanguinis*) were more abundant in the controls. In group PD, a total of 129 taxa showed differential abundances between the RA patients and controls (Supplementary Figure S1B). Of the species level with the logarithmic LDA score > 4, *S. anginosus* and three unidentified species of genera *Actinomyces*, *Fusobacterium*, and *Parvimonas* demonstrated a significant increase in the RA patients, while *Pseudomonas batumici* (*P. batumici*) was the only enriched species found in the controls (the logarithmic LDA score = 5.42).

### 2.5. The Relationships between the Abundance of Discriminative Taxa and the Concentrations of ACPAs in Groups AM and PD

The discriminative microbial species identified in LEfSe (72 and 58 species in groups AM and PD, respectively) were further analyzed to show their correlations with the ACPA levels. In group AM, *Aminipila butyrica* (*A. butyrica*), *Peptococcus simiae* (*P. simiae*), and four other species showed positive correlations with the ACPA levels, which are listed in Table 2. In group PD, 12 species were positively correlated with the ACPA levels, among which were *A. butyrica* and *P. simiae*, also identified in group AM. In group PD, *P. batumici*, displaying the LDA score > 5 between the RA patients and controls by LEfSe, was the only species that showed negative correlations with the ACPA levels.

**Figure 4:**
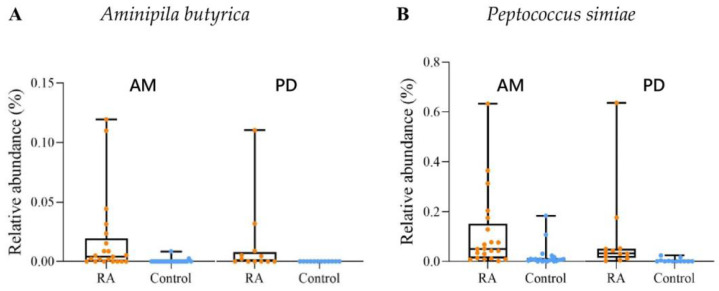
Relative abundances of (**A**) *A. butyrica* and (**B**) *P. simiae* displaying positive correlations to the ACPA levels in both of groups AM and PD. RA, the RA patients in this study; Control, the controls in this study. The data are expressed as median (25th, 75th percentile).

The relative abundances of *A. butyrica* and *P. simiae* are shown in Figure 4. The median of the relative abundance of *A. butyrica* was 0.004% (RA patients) and 0% (controls) of group AM and 0.001% (RA patients) and 0% (controls) of group PD. Of note, only two individuals of the controls in group AM showed colonized *A. butyrica* (Supplementary Table S2). In group PD, *A. butyrica* was entirely absent in all of the control participants (Supplementary Table S3). As for *P. simiae*, the median of the relative abundance was 0.05% (RA patients) and 0.007% (controls) of group AM and 0.03% (RA patients) and 0% (controls) of group PD.

### 2.6. Differences in the Metabolic Pathways between the RA Patients and Controls in Groups AM and PD

PICRUSt2 was used to predict the microbial functional differences based on the 16S rRNA gene sequencing data. In group AM, a total of 107 significantly different MetaCyc metabolic pathways between the RA patients and controls were identified. A total of 54 pathways were overrepresented in the RA patients, including the pathway of arginine, ornithine, and proline interconversion, which involves L-citrulline metabolism (Supplementary Figure S2A). In group PD, only thirteen pathways showed significant differences, and six of them demonstrated enrichment in the RA patients (Supplementary Figure S2B).

## 3. Discussion

The studies with regard to microbiome and RA predominantly focus on Caucasian populations. To the best of the authors’ knowledge, this is the first study to investigate the relationships between subgingival microbiota and RA patients in the Taiwanese population. Significant differences in the subgingival microbial composition between the RA patients and controls in groups AM and PD were observed, and numerous discriminant taxa were found by LEfSe. The Pearson correlation analysis revealed that *A.* *butyrica* and *P. simiae* were the only two species positively correlated to the level of ACPAs in either groups AM or PD. 

The possible roles of *A. butyrica* and *P.* *simiae* in the development of RA were addressed for the first time in the current study. *A. butyrica* demonstrated a 35.2-fold greater abundance in the RA patients of group AM and was not detected in the controls of group PD. The significantly positive correlation among *A. butyrica* and ACPAs further supports the idea that this species might act as a trigger for the induction of ACPAs. *A. butyrica* is an asscharolytic, strictly anaerobic, arginine-decomposing bacterium [14]. According to the whole genome sequence data available in GenBank (accession number NZ_CP048649), *A. butyrica* harbors the gene encoding arginine deiminase, which will degrade arginine and produce substantial amounts of citrulline. The citrullination ability to bacterial proteins may constitute a potent antigen and induce the production of ACPAs, which had been proven in *P. gingivalis* [1,2] and suspected in *Cryptobacterium curtum* [15].

The relative abundance of *P. simiae* was enriched in the RA patients and showed positive correlation to the level of ACPAs in groups AM and PD, suggesting its possible contribution of ACPAs to RA pathogenesis. *P. simiae* is an asscharolytic, strictly anaerobic bacterium, which is similar to the aforementioned *A. butyrica* [16]. Increases of *Peptococcus* have been found in deeper periodontal pockets of chronic periodontitis [17] and enriched in oral squamous cell carcinoma [18]. Cheah et al. reported *Peptococcus* as a disease-associated genus demonstrated a negative correlation with the NRA-H group (controls without RA in a periodontally healthy group) [19], which was in agreement with the present study. However, no data addressed the citrullination ability of this species. It has been reported that LtxA of *A. actinomycetemcomitans*, a pore-forming toxin, would induce cytolysis and neutrophil death called Netosis. The LtxA-mediated Netosis would cause hyperactivation of human PAD2/4, leading to hypercitrullination of specific proteins [2,20]. Moreover, cellular hypercitrullination in neutrophils through the Netosis pathway by using a pore-forming toxin, the RTX family protein, or cytolysins could be expressed by many pathogenic bacteria in addition to *A. actinomycetemcomitans* [20,21,22]. Therefore, we hypothesize that *P. simiae* may possibly increase the expression of human citrullinating PAD enzymes, which are enriched in neutrophils through the Netosis pathway. Human proteins, such as histone, are hypercitrullinated by PADs upon high cellular calcium in neutrophils at the subgingival sites of periodontitis [3]. The hypercitrullinated proteins subsequently become autoantigens to induce the production of ACPAs [2,3]. Further studies are needed to explore the underlying mechanisms.

*Peptostreptococcus stomatis* (*P. stomatis*), an obligate anaerobic and weakly saccharolytic bacterium [23], showed the strongest positive correlation with the level of ACPAs in group AM (the Pearson correlation coefficient = 0.617, *p* < 0.001, Table 2). It raises concerns for *P. stomatis* in triggering ACPA production. Previous studies reported that *P. stomatis* was associated with periodontitis as one of the specific microbial signatures of the subgingival biofilm in periodontitis patients [12,24,25]. Suarez et al. demonstrated that *P. stomatis* was one of pathobionts that is usually underestimated in periodontitis and can trigger immune-inflammatory processes, including autoimmunity in genetically susceptible individuals [26]. It was also reported that *P. stomatis* was related to oral squamous cell carcinoma, gastric cancer, and colorectal cancer [27,28,29,30,31]. Moreover, Tong et al. studied salivary microbiota and showed a positive correlation of the relative abundance of the genus *Peptostreptococcus* to serum ACPA concentration in high-risk individuals [32]. Further studies are needed to explore the ability of *P. stomatis* in inducing ACPA production.

The red complex of periodontal pathogens, *P. gingivalis*, *T. denticola*, and *Tannerella forsythia* (*T. forsythia*) did not show correlations to the levels of ACPAs. Nevertheless, the median of relative abundance in group PD was all higher than that in group PH (*P. gingivalis*, 5.22% vs. 1.31%, *T. denticola*, 0.98% vs. 0.39%, and *T. forsythia*, 1.04% vs. 1.01%) although statistical analysis was not performed due to the fact that the individuals between groups PD and PH were not matched. Moreover, the higher relative abundances of the three periodontal pathogens in the RA patients compared with their matched controls were found in both groups PD or PH although significant increase was only observed in *T. denticola* of group PH (*p* = 0.04, Supplementary Figure S3). Therefore, the 16S rRNA gene sequencing on subgingival microbiota performed in the current study was reliable because the profile of the three periodontal pathogens was in agreement with other previous studies [33,34].

Investigations by LEfSe showed a large amount of discriminant taxa (Supplementary Figure S1). In the species level with logarithmic LDA > 4, five species showed significant changes in relative abundance in groups AM and PD. For the genus *Streptococcus*, *S. anginosus* was overrepresented in the RA patients of groups AM and PD. *S. sanguinis* was also enriched in the RA patients of group PD and displayed positive correlation to the level of ACPAs (Table 2). However, *S. sanguinis* was enriched in the controls of group AM, including those with periodontitis and those who were periodontally healthy. Previous literature also provided controversial results. The overabundance of *Streptococcus* in the deep-periodontal sites of RA or periodontally diseased sites in early RA patients has been reported [11,35], while reduced abundance has been found in RA patients without periodontitis [15,36]. The different findings may be due to the reason that the sampling from the shallow gingival sulcus in periodontally healthy participants was easily affected by environmental factors, such as personal hygiene, diet, and habits. Moreover, *Streptococcus* species are initial colonizers for dental biofilm architecture. They produce CO_2_, lactate, and acetate by consuming O_2_, allowing successful colonization of obligate anaerobes [37,38], such as *A. butyrica* and *P. simiae*, the species responsible for the etiology of RA.

*H. parainfluenzae* and *P. batumici* were the species displaying significant decrease (logarithmic LDA > 4) in the RA patients of groups AM and PD, respectively. *H. parainfluenzae* has been reported to act as an immunomodulatory commensal, which upregulates the expression of PD-L1 and is reduced in saliva microbiota of the patients with Sjögren’s syndrome, an autoimmune epithelitis [39]. *P. batumici* has been proven to secrete an antibiotic called batumin, which will inhibit aminoacyl tRNA synthetase and can blockade the fatty acid biosynthesis pathway on *S. aureus* [40,41]. These two species may contribute to immunomodulation or inhibition of subgingival pathogens, but the mechanism in protection of RA needs to be elucidated.

Our results demonstrated that there was no difference in the alpha diversity of groups AM and PH, but the numbers of the observed bacterial features and the Chao1 index were significantly reduced in the RA patients of group PD (*p* = 0.0375 and 0.0496, Figure 2). It revealed that some periodontal pathogens that caused periodontitis might be responsible for the development of RA. Significantly decreased estimated microbial richness was observed in the ACPA+ at-risk group compared with that in the control group in periodontally healthy sites [35]. 

A significantly distinct clustering of subgingival microbiota between the RA patients and controls was observed in groups AM and PD but not in group PH (Figure 3). It demonstrated there was a different dysbiosis of subgingival microbiota between the RA patients with periodontitis and the controls with periodontitis. Certain periodontal microbes, such as ACPA-promoting bacteria, are related to the development of RA. A great deal of research also displayed a significant difference in subgingival microbial clustering between RA patients and controls [15,42], but some still revealed opposite results [19,36,43,44]. The different results might be explained by different statistical analyses of sequencing [36], diverse sample-pooling methods [42,43,44], and different ratios of the periodontal disease between groups [43]. Cheng’s study demonstrated certain significant differences between early RA patients and controls in periodontally diseased sites and fewer differences between early RA patients and controls in periodontally healthy sites [35]. Our study also demonstrated similar findings.

In the present study, it has proven that lung and related mucosal tissues act as possible sites to trigger an RA-specific autoimmune response [45]. ACPAs were detected in the sputum and were enriched in the bronchoalveolar lavage of patients with early RA, and microscopic lung changes were observed [46,47,48]. In group PH, all of the participants were periodontally healthy, and four of the RA participants were ACPA-positive (Table 1). It implies that the triggering of autoimmunity of RA in group PH may occur in the lungs or other organs rather than in the oral cavity.

In conclusion, there were significant differences in the composition of subgingival microbiota between the RA patients and controls of groups AM and PD. *A. butyrica* and *P. simiae* were the only two species enriched in RA and positively correlated to ACPAs in these two groups. Taken together, *A. butyrica* and *P. simiae*, as periodontal bacteria, play potential roles in the development of RA through the induction of ACPA production by hypercitrullination. Since a causal relationship between the subgingival microbiota and RA etiology cannot be established with a cross-sectional evaluation, further studies are needed to explore the underlying mechanisms.

## 4. Materials and Methods

### 4.1. Study Participants and the Assessment of RA

A total of 30 RA patients and 25 controls were enrolled from the Rheumatology Clinics and the Dental Clinics of Kaohsiung Medical University Hospital (KMUH), respectively. They were diagnosed according to the 2010 RA classification criteria [49]. The exclusion criteria included smokers, those with malignancy or pregnancy, those who were breastfeeding, and those who were under antibiotic treatment within fewer than three months prior to the enrollment. Demographic and behavioral characteristics were collected using questionnaires. The RA patients’ past medical history, diabetes mellitus (DM) statuses, clinical and laboratory data on their RA statuses, and medications were obtained from their medical records. The levels of their serum ACPAs and RF were determined by the Elia CCP kit (Phadia AB, Uppsala, Sweden) and N Latex RF kit (Siemens Healthcare Diagnostics, München, Germany), respectively. The levels of the serum ACPAs and RF from the controls were also determined by the same procedures and technicians as those from the RA patients.

### 4.2. The Subgingival Microbiome Sampling and the Assessments of the Periodontitis Statuses in the RA Patients and Controls

The periodontal statuses of the RA patients and controls were assessed through a standardized, full-mouth periodontal evaluation, including radiographing examinations and/or periodontal assessments, including the probing depth, clinical attachment levels and bleeding on probing, by a periodontist in KMUH. According to the latest Classification of Periodontal Diseases and Conditions, periodontally healthy sites were defined as sites with ≤3 mm probing depth and no bleeding on probing [50]. Periodontitis sites were those with ≥4 mm probing depth and ≥2 mm clinical attachment loss and radiographic bone loss (<15%). The subgingival plaque from all participants was collected at least 3 months after the last teeth cleaning, and the procedure is briefly described below. After having been isolated with cotton rolls and use of suction to remove extra saliva, the subgingival plaque was individually collected by using a sterile endodontic paper point left for 30 s in the deepest gum pocket or the deepest sites of each participant. Up to 5 dental paper-points were pooled from the same site and collected in a 1 mL VMGA transport medium (containing 5% gelatin, 0.005% thioglycolic acid, and 0.005% cysteine) supplied by the Anaerobic and Oral Microbiology Testing Center in Kaohsiung Medical University. The microbial DNA was extracted within four hours after sample collection.

### 4.3. Microbial DNA Extraction, 16S rRNA Gene Sequencing

The subgingival microbial DNA in a 500 µL transport medium was extracted by using the DNeasy PowerBiofilm kit (Qiagen, Hilden, Germany) with a preceding bead beating (45 s; speed: 3450 oscillations/min) and stored at −20 °C. Then, the DNA concentration was determined by a Colibri Microvolume spectrophotometer (Titertek Berthold, Pforzheim, Germany). The 16S rRNA gene of each sample was amplified using the primer pairs 341F (5′-CCTACGGGNGGCWGCAG-3′) and 805R (5′-GACTACHVGGGTATCTAATCC-3′) targeting the V3–V4 region. Library preparation was achieved by the Illumina MiSeq platform generating 300 bp paired-end reads. The raw sequence data were imported into QIIME2 [51], in which paired-end reads were merged and denoised into amplicon sequence variants (ASVs) using the DADA2 plugin [52]. The reads were filtered based on exact matches to the barcode/primer and an average quality score of 30. Due to the issue of sample bleeding between the Illumina MiSeq runs [53], the low-abundance filters (a cutoff threshold of 0.1% of mean frequency 70,651) were applied to reduce the number of spurious ASVs in the data set.

### 4.4. Statistics and Bioinformatic Analysis

The differences between the RA patients and controls were evaluated by the non-parametric Mann–Whitney U-test, independent *t*-test, or chi-square tests with SPSS Version 20.0 (SPSS, Chicago, IL, USA). The significance was determined when *p* < 0.05. 16S rRNA gene sequencing was performed to investigate the composition of the subgingival microbiota. Given that uneven sampling depths can lead to false conclusions in assessing microbiome diversity [54], we rarified the sampling depth of each sample to 11,417 reads at least, which was the lowest number of reads observed across all samples, and where rarefaction curves of all groups had reached an asymptote. The normalized ASV dataset was then used for the analyses of alpha and beta diversity. The number of the observed bacterial features, Chao1, and Shannon indices were used to measure alpha diversity, and their measurement was calculated and compared among the groups by using Kruskal–Wallis tests. For beta diversity, analysis of similarities (ANOSIM) and permutational multivariate analysis of variance (PERMANOVA) with 999 permutations were conducted and evaluated using principal coordinate analyses (PCoA) based on Bray–Curtis dissimilarity [55]. Taxonomy classification of ASVs was performed using the SciKit Learn-based approach [56] searching in the *SILVA* reference database [57] (release v138, trimming to the V3–V4 region, L7 taxonomy), which was subsequently transformed to produce the table of the relative abundance of the taxa for the linear discriminant analysis effect size (LEfSe) [58]. The significantly different features among the taxa were defined with *p* < 0.05 (the factorial Kruskal–Wallis test) and the logarithmic linear discriminant analysis (LDA) score > 2. The Pearson correlation analysis was performed to evaluate the correlation between the level of ACPAs and the discriminant taxa identified in LEfSe. Phylogenetic investigation of communities by reconstruction of observed states 2 (PICRUSt2) [59] was adopted to infer the functional differences between the microbial communities of the groups, and the ASV sequencing reads to functional orthologs were assigned, and the functional pathways were predicted. The results of PICRUSt2 were visualized and tested for statistical significance in STAMP [60]. 

## Figures and Tables

**Table 1:** Clinical characteristics of the RA and control participants.

	AM			PD			PH		
	RA	Control	*p*-Value	RA	Control	*p*-Value	RA	Control	*p*-Value
N	21	21		12	12		6	6	
Age	57.57 ± 8.06	57.19 ± 8.73	0.884	59.00 ± 7.80	58.50 ± 7.86	0.877	54.50 ± 11.66	53.33 ± 11.04	0.862
Female, %	16 (76.19)	16 (76.19)	1	9 (75)	9 (75)	1	5 (83.33)	5 (83.33)	1
Diabetes mellitus, %	4 (19.05)	4 (19.05)	1	2 (16.67)	2 (16.67)	1	1 (16.67)	1 (16.67)	1
Autoantibody status									
RF, positive	17 (80.95)	2 (9.52)	<0.001	11 (91.67)	1 (8.33)	<0.001	5 (83.33)	1 (16.67)	0.021
ACPA, positive	15 (71.43)	0 (0)	<0.001	10 (83.33)	0 (0)	<0.001	4 (66.67)	0 (0)	0.558
ACPA, level	54.50 (3.9, 223.00)	1.00 (0.60, 1.40)	<0.001	97.50 (35.75, 338.50)	0.80 (0.47, 1.18)	<0.001	142.50 (28.20, 191.25)	1.20 (0.85, 1.85)	0.336
Periodontitis, %	14 (66.67)	14 (66.67)	1	12 (100)	12 (100)	1	0 (0)	0 (0)	1

The data are expressed as numbers (percentages) for categorical variables and mean ± SD or median (25th, 75th percentile) for continuous variables as appropriate.

**Table 2:** Correlation between the subgingival microbiota composition and ACPA levels in groups AM and PD.

	Group	
	AM	PD
*Aminipila butyrica*	0.360 (0.019)	0.440 (0.032)
*Peptococcus simiae*	0.464 (0.002)	0.435 (0.033)
*Bacteroides ovatus*	0.313 (0.044)	-
*Bulleidia extructa*	0.316 (0.041)	-
*Genus_Parvimonas* sp.	0.330 (0.033)	-
*Peptostreptococcus stomatis*	0.617 (<0.001)	-
*Genus_Acidovorax* sp.	-	0.440 (0.032)
*Family_Actinomycetaceae* sp.	-	0.441 (0.031)
*Genus_Actinomyces* sp.	-	0.438 (0.032)
*Cloacibacterium haliotis*	-	0.524 (0.009)
*Flexilinea flocculi*	-	0.445 (0.029)
*Genus_Lactobacillus* sp.	-	0.456 (0.025)
*Leptotrichia hofstadii*	-	0.440 (0.031)
*Rivicola pingtungensis*	-	0.416 (0.043)
*Rothia mucilaginosa*	-	0.542 (0.006)
*Streptococcus sanguinis*	-	0.476 (0.019)
*Pseudomonas batumici*	-	−0.436 (0.033)

Correlations are reported as the Pearson correlation coefficient, and *p*-values are given in parentheses. Only species with *p* < 0.05 are listed.

## Data Availability

The 16S rRNA sequencing data files have been deposited in the NCBI Sequence Read Archive. The Bioproject accession number is PRJNA870274.

## Supplementary Materials

The following supporting information can be downloaded at: https://www.mdpi.com/article/10.3390/ijms23179883/s1.
