# Supplementary material for: Subgingival Microbiome in Rheumatoid Arthritis Patients with Periodontitis
Source: Int J Mol Sci. 2022 Aug 31;23(17):9883. doi: 10.3390/ijms23179883 (PMC9456296; doi:10.3390/ijms23179883)
Supplement: Supplementary file 1 [file ijms-23-09883-s001.zip › ijms-1868797-supplementary.pdf]

Supplementary Figure S1

(A) Group AM

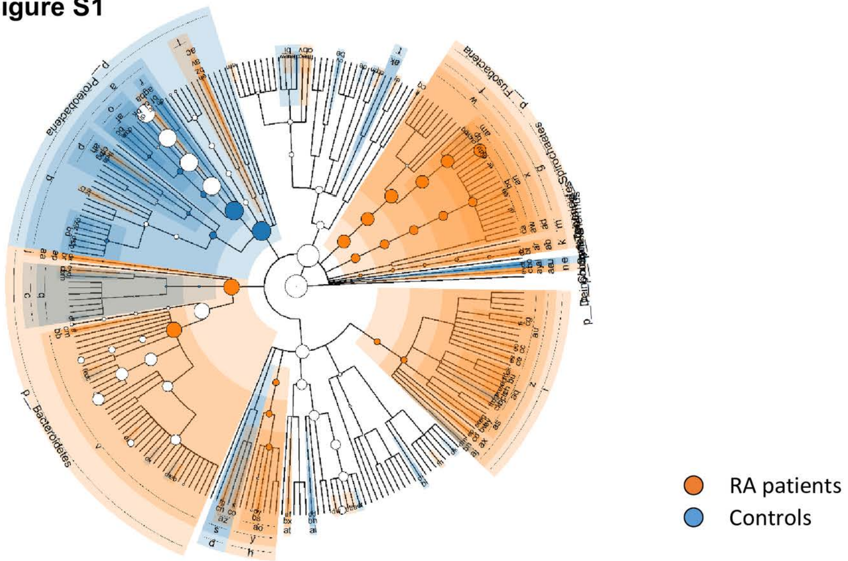

- a: c__Gammaproteobacteria
- aa: o__Phylum_Bacteroidetes sp.
- ab: o__Synergistales
- ac: o__Desulfobacterales
- ad: o__Mycoplasmatales
- ae: o__Anaerolineales
- af: f__Pasteurellaceae
- ag: f__Cardiobacteriaceae
- ah: f__Comamonadaceae
- ai: f__Aerococcaceae
- aj: f__Eubacteriaceae
- ak: f__Bifidobacteriaceae
- al: f__Thermaceae
- am: f__Fusobacteriaceae
- an: f__Peptoniphilaceae
- ao: f__Spirochaetaceae
- ap: f__Clostridiales Family XIII. Incertae Sedis
- aq: f__Phylum_Bacteroidetes sp.
- ar: f__Synergistaceae
- as: f__Peptostreptococcaceae
- at: f__Staphylococcaceae
- au: f__Lachnospiraceae
- av: f__Desulfobulbaceae
- aw: f__Mycoplasmataceae
- ax: f__Peptococcaceae
- ay: f__Anaerolineaceae
- az: f__Erysipelotrichaceae
- b: c__Betaproteobacteria
- ba: f__Moraxellaceae
- bb: f__Bacteroidaceae
- bc: g__Haemophilus
- bd: g__Neisseria
- be: g__Cardiobacterium
- bf: g__Brachybacterium
- bg: g__Family_Comamonadaceae sp
- bh: g__Abiotrophia
- bi: g__Eubacterium
- bj: g__Schaalia
- bk: g__Family_Pasteurellaceae sp.
- bl: g__Family_Selenomonadaceae sp.
- bm: g__Family_Weeksellaceae sp.
- bn: g__Pseudoramibacter
- bo: g__Meiothermus
- bp: g__Family_Leptotrichiaceae sp.
- bq: g__Fusobacterium
- br: g__Treponema
- bs: g__Parvimonas
- bt: g__Phylum_Bacteroidetes sp.
- bu: g__[Eubacterium]
- bv: g__Fretibacterium
- bw: g__Fudania
- bx: g__Peptostreptococcus
- by: g__Staphylococcus
- bz: g__Filifactor
- c: c__Flavobacteriia
- ca: g__Desulfobulbus
- cb: g__Mycoplasma
- cc: g__Johnsonella
- cd: g__Peptococcus
- ce: g__Family_Clostridiales Family XIII. Incertae Sedis sp.
- cf: g__Catonella
- cg: g__Flexilinea
- ch: g__Oribacterium
- ci: g__Mogibacterium
- cj: g__Rivicola
- ck: g__Cloacibacterium
- cl: g__Aminipila
- cm: g__Moraxella
- cn: g__Bacteroides
- co: g__Solobacterium
- cp: g__Bulleidia
- cq: g__Acidovorax
- cr: g__Family_Actinomycetaceae sp.
- cs: g__Shuttleworthia
- ct: s__parainfluenzae ATCC 33392
- cu: s__sanguinis SK1 = NCTC 7863

- cv: s__nucleatum subsp. polymorphum ATCC 10953
- cw: s__elongata subsp. nitroreducens
- cx: s__Genus_Neisseria sp.
- cy: s__Genus_Brachybacterium sp.
- cz: s__nucleatum subsp. vincentii ATCC 49256
- d: c__Erysipelotrichia
- da: s__Family_Comamonadaceae sp.
- db: s__catoniae ATCC 51270
- dc: s__melaninogenica ATCC 25845
- dd: s__defectiva
- de: s__Cardiobacterium_hominis
- df: s__durum
- dg: s__ochracea
- dh: s__nodatum ATCC 33099
- di: s__Family_Pasteurellaceae sp.
- dj: s__cardiffensis
- dk: s__Family_Selenomonadaceae sp.
- dl: s__noxia
- dm: s__shayeganii 871
- dn: s__rubra
- do: s__Family_Weeksellaceae sp.
- dp: s__alactolyticus ATCC 23263
- dq: s__Genus_Haemophilus sp.
- dr: s__periodonticum
- ds: s__pallens ATCC 700821
- dt: s__perflava
- du: s__silvanus
- dv: s__Family_Leptotrichiaceae sp.
- dw: s__Genus_Fusobacterium sp.
- dx: s__Treponema_denticola
- dy: s__anginosus SK52 = DSM 20563
- dz: s__Genus_Parvimonas sp.
- e: c__Deinococci
- ea: s__pleuritidis
- eb: s__Phylum_Bacteroidetes sp.
- ec: s__fastidiosum
- ed: s__medium
- ee: s__jinshanensis
- ef: s__brachy
- eg: s__Peptostreptococcus_stomatis
- eh: s__Genus_Staphylococcus sp.
- ei: s__alocis
- ej: s__uli DSM 7084
- ek: s__fusca JCM 17724
- el: s__oligotrophicus
- em: s__viscosus
- en: s__Genus_Treponema sp.
- eo: s__saphenum
- ep: s__ignava ATCC 51276
- eq: s__simiae
- er: s__Family_Clostridiales Family XIII. Incertae Sedis sp.
- es: s__gordonii
- et: s__georgiae
- eu: s__propionica
- ev: s__morbi ATCC 51271
- ew: s__socranskii subsp. buccale
- ex: s__flocculi
- ey: s__meyeri
- ez: s__timidum
- f: c__Fusobacteriia
- fa: s__haliotis
- fb: s__pingtungensis
- fc: s__butyrica
- fd: s__mutans
- fe: s__osloensis
- ff: s__ovatus
- fg: s__vulgatus
- fh: s__infirmum
- fi: s__Genus_Acidovorax sp.
- fj: s__moorei
- fk: s__extructa
- fl: s__Genus_Oribacterium sp.
- fm: s__dianae
- g: c__Tissierellia
- h: c__Spirochaetia
- i: c__Clostridia

- j: c__Phylum_Bacteroidetes sp.
- k: c__Synergistia
- l: c__Mollicutes
- m: c__Deltaproteobacteria
- n: c__Anaerolineae
- o: o__Pasteurellales
- p: o__Burkholderiales
- q: o__Flavobacteriales
- r: o__Cardiobacteriales
- s: o__Erysipelotrichales
- t: o__Bifidobacteriales
- u: o__Thermales
- v: o__Bacteroidales
- w: o__Fusobacteriales
- x: o__Tissierellales
- y: o__Spirochaetales
- z: o__Eubacteriales

Supplementary Figure S1

(B) Group PD

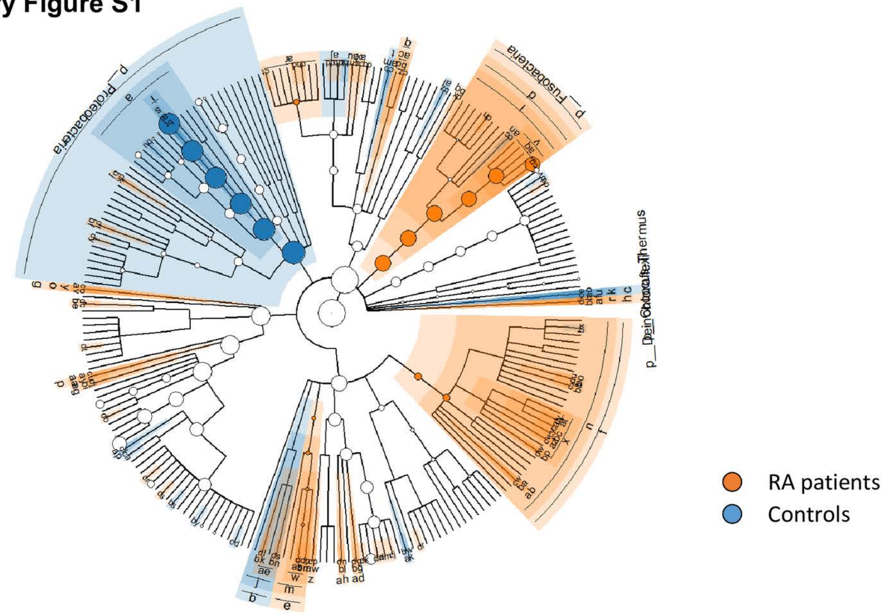

- a: c__Gammaproteobacteria
- aa: f__Phylum_Bacteroidetes sp.
- ab: f__Propionibacteriaceae
- ac: f__Marinilabiliaceae
- ad: f__Peptococcaceae
- ae: f__Erysipelotrichaceae
- af: f__Aerococcaceae
- ag: f__Lactobacillaceae
- ah: f__Bacteroidaceae
- ai: f__Anaerolineaceae
- aj: g__Pseudomonas
- ak: g__Schaalia
- al: g__Family_Selenomonadaceae sp.
- am: g__Lancefieldella
- an: g__Brachybacterium
- ao: g__Meiothermus
- ap: g__Family_Leptotrichiaceae sp.
- aq: g__Desulfomicrobium
- ar: g__Fusobacterium
- as: g__Actinomyces
- at: g__Parvimonas
- au: g__Fudania
- av: g__[Eubacterium]
- aw: g__Staphylococcus
- ax: g__Family_Actinomycetaceae sp.
- ay: g__Phylum_Bacteroidetes sp.
- az: g__Arachnia
- b: c__Erysipelotrichia
- ba: g__Johnsonella
- bb: g__Family_Marinilabiliaceae sp.
- bc: g__Peptococcus
- bd: g__Family_Clostridiales Family XIII. Incertae Sedis sp
- be: g__Mogibacterium
- bf: g__Cloacibacterium
- bg: g__Abiotrophia
- bh: g__Lactobacillus
- bi: g__Rivicola
- bj: g__Bacteroides
- bk: g__Flexilinea
- bl: g__Acidovorax
- bm: g__Solobacterium
- bn: g__Bulleidia
- bo: g__Peptoniphilus
- bp: g__Lacrimispora
- bq: g__Aminipila
- br: s__batumici
- bs: s__melaninogenica ATCC 25845
- bt: s__cardiffensis
- bu: s__Family_Selenomonadaceae sp.
- bv: s__Genus_Haemophilus sp.
- bw: s__succinifaciens DSM 2489
- bx: s__saburreum DSM 3986
- by: s__rimae
- bz: s__pallens ATCC 700821
- c: c__Deinococci
- ca: s__rava
- cb: s__Genus_Brachybacterium sp.

- cc: s__salivae
- cd: s__silvanus
- ce: s__Family_Leptotrichiaceae sp.
- cf: s__orale
- cg: s__Genus_Fusobacterium sp.
- ch: s__Genus_Actinomyces sp.
- ci: s__anginosus SK52 = DSM 20563
- cj: s__Genus_Parvimonas sp.
- ck: s__sanguinis SK1 = NCTC 7863
- cl: s__jinshanensis
- cm: s__gerencseriae
- cn: s__brachy
- co: s__Genus_Staphylococcus sp.
- cp: s__Family_Actinomycetaceae sp.
- cq: s__loescheii
- cr: s__fusca JCM 17724
- cs: s__Phylum_Bacteroidetes sp.
- ct: s__viscosus
- cu: s__propionica
- cv: s__ignava ATCC 51276
- cw: s__gordonii
- cx: s__Family_Marinilabiliaceae sp.
- cy: s__simiae
- cz: s__Family_Clostridiales Family XIII. Incertae Sedis sp.
- d: c__Fusobacteriia
- da: s__georgiae
- db: s__meyeri
- dc: s__timidum
- dd: s__haliotis
- de: s__socranskii subsp. buccale
- df: s__defectiva
- dg: s__Genus_Lactobacillus sp.
- dh: s__pingtungensis
- di: s__hofstadii
- dj: s__vulgatus
- dk: s__ovatus
- dl: s__mutans
- dm: s__flocculi
- dn: s__Genus_Acidovorax sp.
- do: s__moorei
- dp: s__infirmum
- dq: s__Genus_Peptoniphilus sp.
- dr: s__extructa
- ds: s__artemidis
- dt: s__haemolytica
- du: s__Genus_Lacrimispora sp.
- dv: s__mucilaginosa
- dw: s__butyrica
- dx: s__halliae
- e: c__Tissierellia
- f: c__Clostridia
- g: c__Phylum_Bacteroidetes sp.
- h: c__Anaerolineae
- i: o__Pseudomonadales
- j: o__Erysipelotrichales
- k: o__Thermales
- l: o__Fusobacteriales

- m: o__Tissierellales
- n: o__Eubacteriales
- o: o__Phylum_Bacteroidetes sp.
- p: o__Propionibacteriales
- q: o__Marinilabiliales
- r: o__Anaerolineales
- s: f__Pseudomonadaceae
- t: f__Dermabacteraceae
- u: f__Thermaceae
- v: f__Desulfomicrobiaceae
- w: f__Fusobacteriaceae
- x: f__Peptoniphilaceae
- y: f__Clostridiales Family XIII. Incertae Sedis
- z: f__Staphylococcaceae

Supplementary Figure S1. Differences in the relative abundances of subgingival microbiota between the RA patients and controls were estimated by LEfSe and presented as a cladogram. (A) Group AM. (B) Group PD. The black circles from inside to outside represent the phylum, class, order, family, and genus. The orange dots and the blue dots represent the significantly enriched microbiota in the RA patients and controls, respectively. The diameter of each dot is proportional to its relative abundance.

Supplementary Figure S2

(A) Group AM

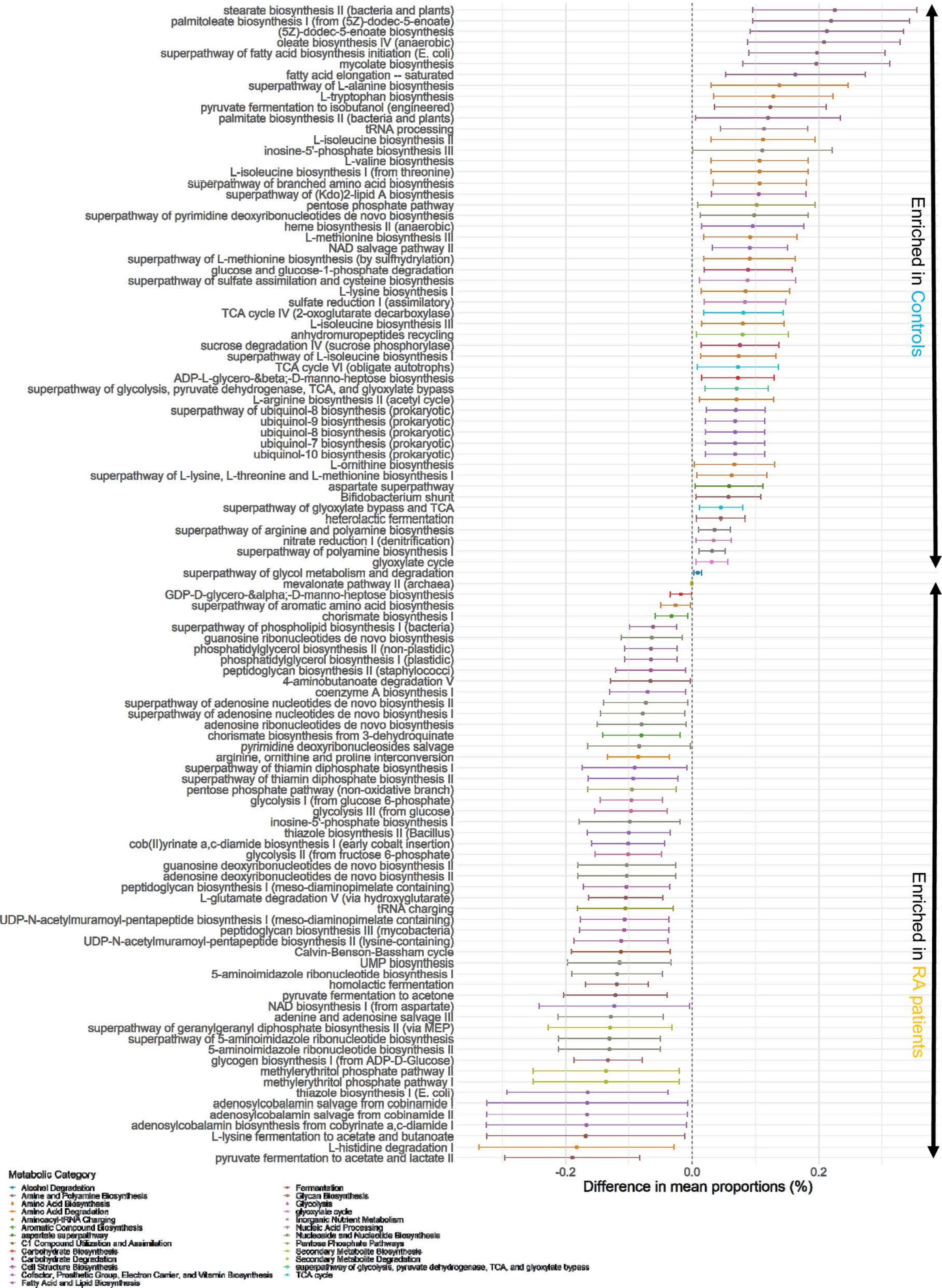

Supplementary Figure S2

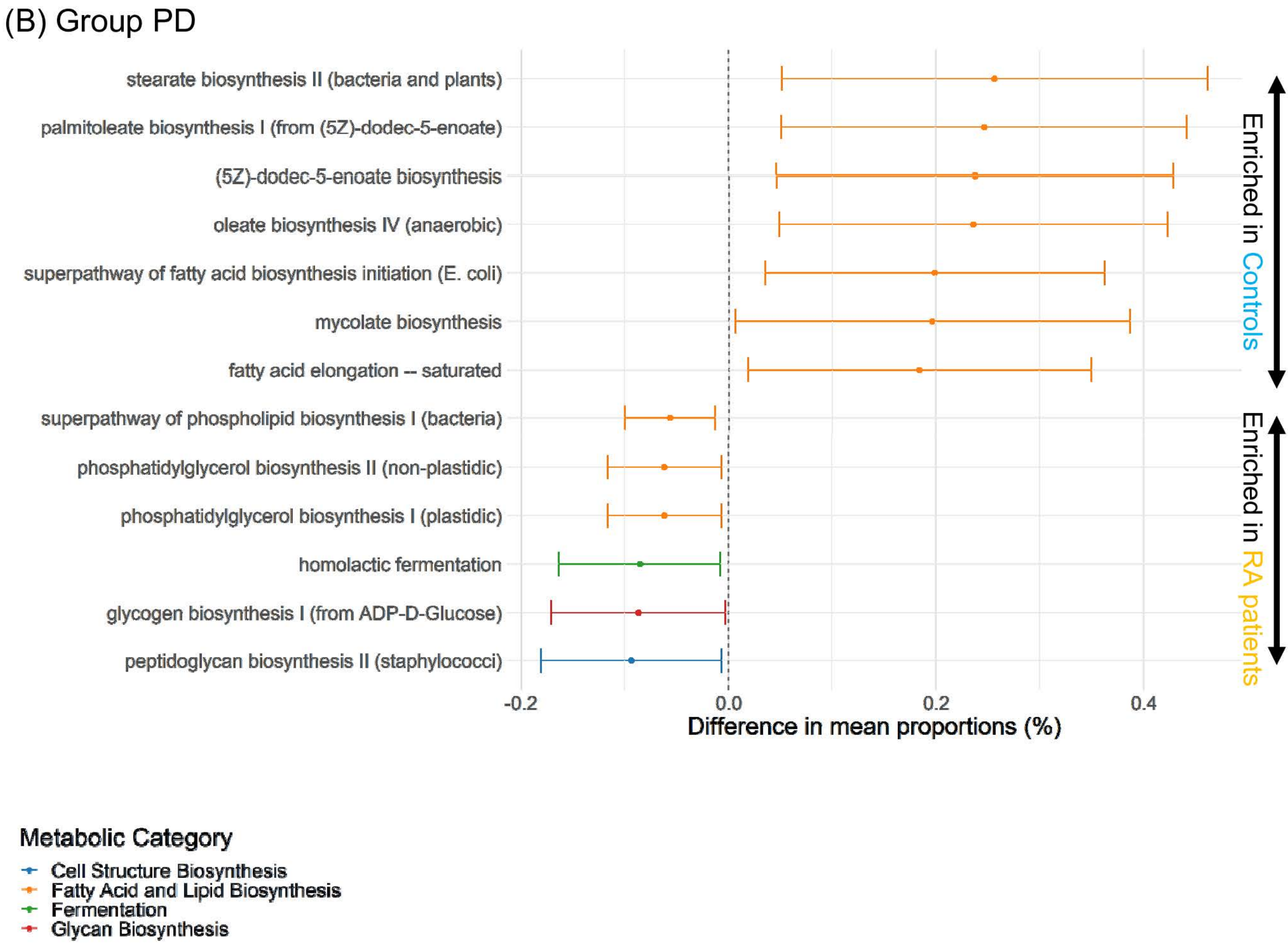

Supplementary Figure S2. Predicted functional differences between the microbiomes of the RA patients and controls assessed by PICRUSt2. (A) Group AM. (B) Group PD. The values of the mean proportion (%) represented in the graph were obtained by STAMP. The level of the classification hierarchy for metabolic pathways are indicated in different colors.

Supplementary Figure S3

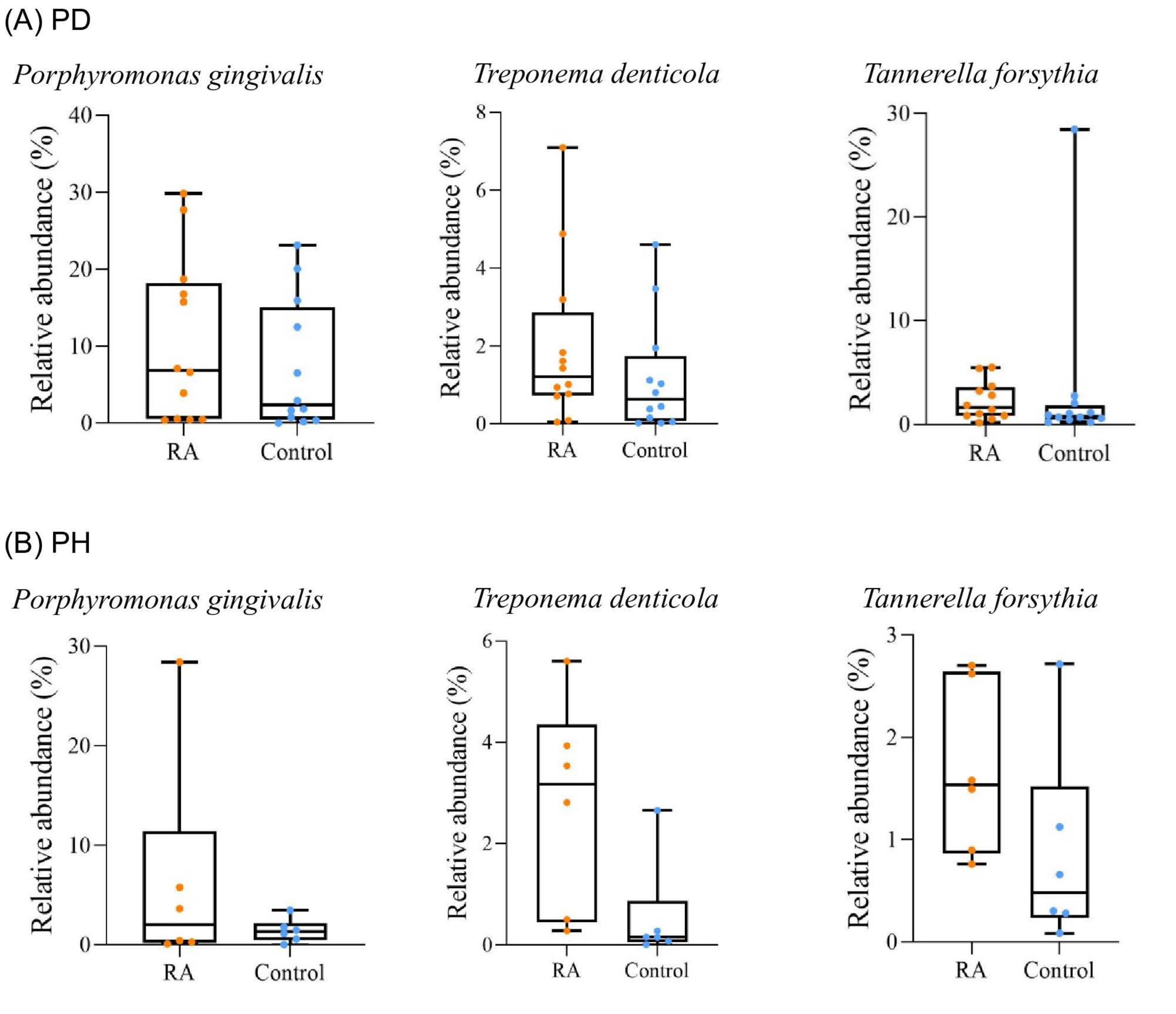

Supplementary Figure S3. Relative abundances of *Porphyromonas gingivalis*, *Treponema denticola*, *Tannerella forsythia* in groups (A) PD and (B) PH. The data are expressed as median (25th, 75th percentile).

Supplementary Table S1. Detailed information of the 55 participants

ID	Type	Age	Sex	RF	ACPA	ACPA (U/ml)	Periodontitis	DM	HbA1c %	Groups		
										AM	PD	PH
N01	Control	51	M	N	N	0.4	Yes	No	-	-	-	-
N02	Control	62	M	P	N	0.6	No	No	-	O	-	O
N03	Control	68	F	N	N	0.4	No	Yes	6.6	O	-	-
N10	Control	53	F	N	N	0.4	Yes	No	-	O	O	-
N11	Control	48	F	P	N	0.4	Yes	No	-	O	O	-
N13	Control	64	F	N	N	0.8	Yes	No	-	O	O	-
N14	Control	38	F	N	N	3.6	Yes	No	-	O	-	-
N15	Control	62	F	N	P	15	Yes	Yes	7.7	-	-	-
N16	Control	64	F	N	N	0.8	Yes	Yes	6	O	O	-
N17	Control	55	F	N	N	2	No	No	-	O	-	O
N18	Control	69	M	N	N	1	Yes	No	-	O	O	-
N19	Control	66	F	P	P	397	Yes	No	-	-	-	-
N21	Control	63	F	N	N	2.1	Yes	No	-	O	O	-
N22	Control	43	F	N	N	1.4	No	No	-	O	-	O
N23	Control	69	M	N	N	0.8	Yes	Yes	7.1	O	O	-
N24	Control	67	F	N	N	2.1	No	No	-	O	-	O
N25	Control	54	M	N	N	1	No	No	-	O	-	-
N26	Control	57	F	N	N	1.1	Yes	Yes	6.6	O	-	-
N30	Control	56	M	N	N	0.4	Yes	No	-	O	O	-
N31	Control	38	F	N	N	0.8	No	Yes	6.7	-	-	O
N33	Control	56	F	N	N	1.4	Yes	No	-	O	O	-
N34	Control	47	F	N	N	1.1	Yes	No	-	O	O	-
N35	Control	55	F	N	N	1	No	No	-	O	-	O
N36	Control	50	F	N	N	0.5	Yes	No	-	O	O	-
N37	Control	63	F	N	N	1.9	Yes	No	-	O	O	-
RA01	RA	47	F	N	N	1.8	Yes	No	-	O	O	-
RA02	RA	63	F	N	P	18.5	No	No	-	O	-	-
RA03	RA	46	F	P	P	23	Yes	Yes	6.8	-	-	-
RA04	RA	58	M	P	P	32.3	Yes	No	-	O	-	-
RA05	RA	67	F	P	N	1.3	Yes	No	-	-	-	-
RA06	RA	56	F	P	P	71	Yes	No	-	O	-	-
RA08	RA	59	F	P	P	216	No	No	-	-	-	-
RA09	RA	51	F	P	P	197	No	No	-	-	-	O
RA10	RA	52	F	P	P	338	Yes	No	-	O	O	-
RA11	RA	71	M	P	P	59	Yes	No	-	-	O	-
RA12	RA	65	F	P	P	340	Yes	No	-	-	O	-
RA13	RA	65	M	P	P	37	Yes	Yes	6.2	O	O	-
RA14	RA	66	F	P	P	49	Yes	No	-	-	-	-
RA15	RA	64	F	P	P	340	Yes	No	-	O	O	-
RA16	RA	55	F	P	N	0.6	No	Yes	6.6	O	-	O
RA18	RA	58	M	P	P	32	Yes	No	-	O	O	-
RA19	RA	67	F	P	P	174	No	No	-	O	-	O
RA20	RA	51	F	P	P	212	Yes	No	-	-	O	-
RA21	RA	49	F	P	N	1	No	No	-	O	-	-
RA23	RA	65	M	P	P	340	Yes	No	-	O	-	-
RA24	RA	58	F	P	P	223	Yes	No	-	O	-	-
RA25	RA	55	F	P	P	136	Yes	No	-	O	O	-

RA26	RA	69	M	P	P	111	No	No	-	O	-	O
RA27	RA	45	F	P	P	340	No	No	-	O	-	O
RA28	RA	66	F	P	P	340	Yes	Yes	6.1	O	O	-
RA29	RA	50	F	P	P	54.5	Yes	No	-	O	O	-
RA30	RA	64	F	P	N	0.7	Yes	No	-	O	O	-
RA31	RA	40	F	N	N	0.5	No	No	-	O	-	O
RA37	RA	63	F	N	N	3.9	Yes	Yes	6	O	-	-
RA38	RA	66	F	P	P	340	Yes	No	-	-	-	-

M, male; F, female; P, positive; N, negative.

Supplementary Table S2. Relative abundances of *Aminipila butyrica* and *Peptococcus simiae* of the 42 individuals in group AM

ID	Type	Age	Sex	ACPA (U/ml)	Periodontitis	*A. butyrica* (%)	*P. simiae* (%)
N02	Control	62	M	0.6	H	0.002426	0.007278
N03	Control	68	F	0.4	H	0.008410	0.107646
N10	Control	53	F	0.4	P	0	0.017087
N11	Control	48	F	0.4	P	0	0.002082
N13	Control	64	F	0.8	P	0	0.023671
N14	Control	38	F	3.6	P	0	0.183764
N16	Control	64	F	0.8	P	0	0.004175
N17	Control	55	F	2	H	0	0
N18	Control	69	M	1	P	0	0
N21	Control	63	F	2.1	P	0	0.010342
N22	Control	43	F	1.4	H	0	0
N23	Control	69	M	0.8	P	0	0.008320
N24	Control	67	F	2.1	H	0	0
N25	Control	54	M	1	H	0	0.007011
N26	Control	57	F	1.1	P	0	0.003548
N30	Control	56	M	0.4	P	0	0.031323
N33	Control	56	F	1.4	P	0	0.009258
N34	Control	47	F	1.1	P	0	0.004395
N35	Control	55	F	1	H	0	0.005676
N36	Control	50	F	0.5	P	0	0.007675
N37	Control	63	F	1.9	P	0	0.009574
RA01	RA	47	F	1.8	P	0.015371	0.034585
RA02	RA	63	F	18.5	H	0.119497	0.128832
RA04	RA	58	M	32.3	P	0.004884	0.068371
RA06	RA	56	F	71	P	0.001995	0.313186
RA10	RA	52	F	338	P	0.044393	0.632595
RA13	RA	65	M	37	P	0.008747	0.174948
RA15	RA	64	F	340	P	0.110007	0.050342
RA16	RA	55	F	0.6	H	0.008657	0.008657
RA18	RA	58	M	32	P	0.003990	0.001995
RA19	RA	67	F	174	H	0	0.365148
RA21	RA	49	F	1	H	0.001822	0.076517
RA23	RA	65	M	340	P	0.023527	0.204506
RA24	RA	58	F	223	P	0	0.013996
RA25	RA	55	F	136	P	0	0.030749
RA26	RA	69	M	111	H	0	0.008720
RA27	RA	45	F	340	H	0.002211	0.077369
RA28	RA	66	F	340	P	0.005206	0.012146
RA29	RA	50	F	54.5	P	0.031559	0.043393
RA30	RA	64	F	0.7	P	0	0.051263
RA31	RA	40	F	0.5	H	0	0.007596
RA37	RA	63	F	3.9	P	0	0.042715

M, male; F, female; P, periodontitis; H, healthy periodontal condition.

Supplementary Table S3. Relative abundances of *Aminipila butyrica* and *Peptococcus simiae* of the 24 individuals in group PD

ID	Type	Age	Sex	ACPA (U/ml)	Periodontitis	*A. butyrica* (%)	*P. simiae* (%)
N10	Control	53	F	0.4	P	0	0.017236
N11	Control	48	F	0.4	P	0	0.002084
N13	Control	64	F	0.8	P	0	0.023817
N16	Control	64	F	0.8	P	0	0.004187
N18	Control	69	M	1	P	0	0
N21	Control	63	F	2.1	P	0	0
N23	Control	69	M	0.8	P	0	0
N30	Control	56	M	0.4	P	0	0
N33	Control	56	F	1.4	P	0	0
N34	Control	47	F	1.1	P	0	0
N36	Control	50	F	0.5	P	0	0
N37	Control	63	F	1.9	P	0	0
RA01	RA	47	F	1.8	P	0	0.034870
RA10	RA	52	F	338	P	0	0.635735
RA11	RA	71	M	59	P	0	0.030445
RA12	RA	65	F	340	P	0.004497	0.020236
RA13	RA	65	M	37	P	0.008809	0.176170
RA15	RA	64	F	340	P	0.110313	0.050482
RA18	RA	58	M	32	P	0.002006	0.002006
RA20	RA	51	F	212	P	0	0.004640
RA25	RA	55	F	136	P	0	0.030996
RA28	RA	66	F	340	P	0.005232	0.012207
RA29	RA	50	F	54.5	P	0.031885	0.043841
RA30	RA	64	F	0.7	P	0	0.052007

M, male; F, female; P, periodontitis; H, healthy periodontal condition.
